# Unravelling the Therapeutic Potential of Cysteine for Cardiovascular Health

**DOI:** 10.3390/ijms27188412

**Published:** 2026-09-21

**Authors:** Satirah Zainalabidin, Gayaatri Ravindran, Muhamad Adib Abdul Ghani, Aiman Zakwan Azman, Chai Yi Ping, Asna Amanina Ahmad, Nur Liyana Mohammed Yusof, Fatin Farhana Jubaidi

**Affiliations:** 1 Center for Toxicology and Health Risk Studies, Faculty of Health Sciences, Universiti Kebangsaan Malaysia, Kuala Lumpur 50300, Malaysia; satirah@ukm.edu.my (S.Z.); ravindrangayaatri@gmail.com (G.R.); p117482@siswa.ukm.edu.my (M.A.A.G.); aimanzakwanazman@gmail.com (A.Z.A.);; 2 Cardiovascular and Pulmonary (CardioResep) Research Group, Universiti Kebangsaan Malaysia, Bangi 43600, Malaysia; nurliyanamyusof@ukm.edu.my; 3 Department of Pharmacology, Faculty of Medicine, Universiti Kebangsaan Malaysia, Kuala Lumpur 56000, Malaysia; 4 Department of Physiology, Faculty of Medicine, Universiti Kebangsaan Malaysia, Kuala Lumpur 56000, Malaysia

**Keywords:** cysteine, cardiovascular disease, oxidative stress, Nrf2 pathway, glutathione, nutraceuticals, hydrogen sulfide

## Abstract

Cardiovascular diseases (CVDs) are a major global health concern, representing a leading cause of death and imposing a significant financial burden on public healthcare systems worldwide. They raise national healthcare expenditure into trillions of dollars globally. The development and progression of CVDs are heavily driven by oxidative stress, inflammation, lipid dysregulation, and endothelial dysfunction. Recent studies have highlighted the potent antioxidant and anti-inflammatory properties of cysteine-containing compounds, suggesting their role in mitigating cardiac diseases. This review explores the cardioprotective mechanisms of cysteine compounds such as N-acetyl cysteine, S-allyl cysteine, S-ethyl cysteine, and S-1-propenyl cysteine, emphasizing their roles in redox homeostasis, endothelial function preservation, mitochondrial protection, and inflammation attenuation. Specifically, we discuss how these compounds exert beneficial effects by serving as precursors to glutathione, acting as hydrogen sulfide (H_2_S) donors, and targeting the Nrf2 pathway through the modulation of Keap1. Ultimately, this review highlights the potential of cysteine-based compounds as candidate adjunctive strategies for cardiovascular disease management. By effectively reducing oxidative stress and limiting inflammatory cascades, incorporating these compounds into dietary interventions could play a transformative role in minimizing myocardial damage and improving patient outcomes.

## 1. Introduction

The Global Burden of Disease study estimated 422.7 million cases of cardiovascular disease (CVD) and 17.92 million cardiovascular deaths worldwide in 2015 [1]. CVDs have become a leading global health concern, with a significant increase in prevalence worldwide [2] These diseases encompass a range of conditions affecting the heart and blood vessels, such as heart disease, ischemic heart disease, vascular disorders, and metabolic conditions, such as hypertension and diabetes, that contribute to cardiovascular risks [3]. The growing burden of CVDs is linked to factors such as urbanization, sedentary lifestyles, unhealthy diets, and increasing rates of obesity and stress. Combined with an increasingly aging population, these factors have led to a higher morbidity and mortality rates associated with cardiovascular complications [4].

More recent epidemiological updates indicate that this upward trajectory continues persistently, with the worldwide prevalence of CVD now surging past 523 million cases [3]. This overwhelming global trend is mirrored closely in rapidly developing nations. In Malaysia particularly, CVDs have emerged as a critical public health issue where CVD accounts for a substantial proportion of deaths and hospitalizations. CVD is the leading cause of death, contributing to 21.65% of fatalities in government hospitals and 23.79% in private hospitals in 2018 [5]. The prevalence of CVD has shown a significant upward trend, with total deaths increasing from 15.4% in 2006 to 25.4% in 2010. Factors contributing to these worrying statistics include high rates of diabetes, hypertension, and dyslipidemia, as well as the widespread consumption of diets high in saturated fats and sugars [6].

CVDs arise primarily due to the development of atherosclerosis, a condition in which plaques build up in blood vessels, often exacerbated by genetic predisposition, metabolic disorders, and aging [7]. Additionally, the prevalence of smoking and limited physical activity further exacerbate this risk [8]. Smoking damages the blood vessels and promotes clot formation [9,10], while a sedentary lifestyle and unhealthy eating habits lead to obesity, hypertension, and dyslipidemia, thus creating a vicious cycle of cardiovascular risk [11].

Consequently, the impact of CVDs extends beyond individual health and imposes a significant burden on governments and societies [4]. The high costs of treatment, hospitalizations, and medications put significant strain on the healthcare systems, while loss of productivity due to premature deaths and disabilities undermines economic growth. This burden underscores the urgency of reducing CVD mortality and morbidity rates. Preventative measures such as promoting healthier lifestyles and improving early detection are not only vital for saving lives but also for alleviating the socioeconomic strain caused by healthcare expenditures and diminished quality of life across communities [12]. Hence, addressing CVDs is essential for fostering healthier and more productive societies and achieving long-term economic and public health benefits.

Living with CVD often involves physical limitations, emotional strain, and a reduced sense of well-being, making the enhancement of patient quality of life a critical clinical target. While pharmaceutical drugs remain central to CVD management by effectively controlling blood pressure, cholesterol, and clot formation [13], their adverse side effects can sometimes diminish overall well-being [14,15]. Consequently, there is a pressing need for innovative treatments that balance cardiovascular efficacy with long-term safety. One highly promising avenue is the use of nutraceuticals, particularly cysteine-based compounds, which possess powerful antioxidant and anti-inflammatory properties [16]. By directly mitigating oxidative stress and inflammation which are the two primary drivers of atherosclerosis and related CVD complications, preclinical studies suggest cysteine-based nutraceuticals offer a promising, safe and targeted strategy to improve cardiovascular outcomes, though robust clinical validation remains necessary.

While William Hyde Wollaston first discovered cystine in 1810, it was Eugen Baumann who later isolated cysteine, the monomeric form of cystine in 1884 [17]. Baumann discovered that cysteine is a breakdown of cystine and plays a significant role in biochemical processes that involve protein structure and function due to its sulfur-containing thiol (-SH) group, which can form disulfide bonds that are important in defining the structure of many proteins, stabilizing extracellular proteins, conferring proteolytic resistance, and enabling the catalytic properties of enzymes [18]. Moreover, because of the presence of the thiol side chain, cysteine is an important source of sulfur in human metabolism, especially for infants, the elderly, and individuals with metabolic diseases. Because our body can synthesize cysteine in the liver from methionine, it is classified as a semi-essential amino acid. Beyond serving as a building block for proteins, cysteine is also vital for cellular defense, as it acts as a precursor of glutathione (GSH), which is one of the major endogenous antioxidants [19]. GSH stimulates leukotriene production, a key component of the body’s defense system, while also inhibiting inflammatory processes and enhancing immune function [20]. However, other than its potent antioxidant properties, cysteine’s role in cardiovascular health remains unclear.

### Literature Search Strategy

To elucidate this role and review the current evidence regarding the cardioprotective mechanisms of cysteine, a comprehensive literature search was conducted across PubMed, Scopus, and Web of Science databases for studies published up to January 2026. Search terms included combinations of (“cysteine” OR “N-acetylcysteine” OR “S-allyl cysteine” OR “S-1-propenyl cysteine” OR “S-ethyl cysteine”) AND (“cardiovascular disease” OR “myocardial infarction” OR “ischemia-reperfusion injury” OR “hypertension” OR “atherosclerosis” OR “endothelial dysfunction” OR “oxidative stress”). Peer-reviewed, English-language studies evaluating cardiovascular mechanisms in in vitro, in vivo, or clinical settings were included. Non-cardiovascular or purely non-mechanistic studies were excluded unless utilized for supportive biochemical context.

## 2. Key Mechanisms in the Pathophysiology of Cardiovascular Diseases

To further discuss the potential role of cysteine in mitigating CVDs, deeper understanding of the underlying mechanisms involved in their development and progression is of the utmost importance. CVDs are complex and multifactorial by nature, influenced by genetic, metabolic, and environmental factors [21]. Their development and progression are driven by oxidative stress, inflammation, lipid dysregulation, cell deaths (including apoptosis and necrosis), and mitochondrial impairment, all of which contribute to structural and functional deterioration of the heart and vessels [22,23]. A clear understanding of these mechanisms provides the foundation for evaluating how cysteine may exert its cardioprotective effects.

### 2.1. Oxidative Stress

The mitochondrial respiratory chain is a major intracellular source of reactive oxygen species (ROS) [24]. As ROS generation is closely linked to oxygen utilization, cells with high oxygen demand such as neurons, cardiomyocytes and hepatocytes are particularly susceptible to oxidative stress [25,26,27]. Cardiac myocytes, which rely heavily on oxidative metabolism, contain an abundance of mitochondria and elevated levels of respiratory chain components per milligram of mitochondrial protein. To counteract potential oxidative injury, cardiomyocytes are equipped with an array of enzymatic ROS-scavenging systems to neutralize the damaging effects of ROS. These includes enzymatic antioxidants such as superoxide dismutase, glutathione peroxidase and catalase, as well as non-enzymatic antioxidants like coenzyme Q10 and glutathione [28,29]. Moreover, mitochondria themselves have the capacity to repair oxidative damage through enzyme systems such as selenoprotein glutathione peroxidase 4 (GPx4), previously known as phospholipid hydroperoxide glutathione peroxidase (PHGP). This selenium-dependent enzyme directly reduces peroxidized acyl groups within phospholipids [30]. Nevertheless, under pathological conditions, these protective mechanisms may become insufficient, resulting in mitochondrial dysfunction that leaves the cardiac myocytes susceptible to oxidative injury. Mitochondrial dysfunction, especially within cardiomyocytes, contributes to the onset and progression of cardiovascular diseases, leading to conditions such as heart failure, cardiomyopathy, and ischemia/reperfusion injury [31].

Historically, ROS have been viewed solely as the detrimental byproduct of cellular metabolism. However, current cardiovascular paradigms recognize the distinct duality of ROS, which can dictate both physiological and pathological outcomes based on their concentration, duration and cellular context [24,28]. At low to moderate levels, mitochondrial ROS act as essential secondary messengers. They mediate important cellular responses including adaptation to hypoxia, regulation of vascular tone, and the activation of endogenous survival pathway, which is central to ischemic preconditioning [24,32].

Conversely, when ROS production overwhelms the cardiomyocytes’ antioxidant defenses, it becomes severely maladaptive [28]. This shift is most pronounced during myocardial ischemia–reperfusion injury, whereby during the ischemic phase, oxygen deprivation severely affects mitochondrial respiratory chain complexes. Upon perfusion, the sudden influx of oxygen triggers a massive, uncontrolled burst of mitochondrial ROS [33]. This pathological surge induces extensive lipid peroxidation, compromises sarcolemmal integrity, and triggers the opening of mPTP, ultimately resulting in irreversible cardiomyocyte apoptosis and necrosis [31,32].

Indeed, cardiac pathologies are predominantly driven by the overproduction of ROS, particularly superoxide (O_2_^−^), hydrogen peroxide (H_2_O_2_), and the hydroxyl radical (•OH) [32]. During cardiac events such as reperfusion following myocardial ischemia, the sudden reintroduction of oxygen generates free radicals that damage cellular membranes, weaken the sarcolemma, and impair enzymatic activity. Free radicals also trigger the endothelium to produce platelet-activating factor (PAF), reducing cardiac function, increasing vascular resistance, and recruiting neutrophils, which amplifies ROS production and reperfusion damage [33,34]. Nitric oxide, which is quenched by ROS, forms peroxynitrite, exacerbating endothelial damage and microvascular dysfunction. Impaired endothelium-dependent vasodilation and enhanced vasoconstrictor production lead to coronary vasoconstriction and reduced blood flow. The lack of antioxidant enzymes further worsens heart dysfunction caused by ROS [35].

### 2.2. Inflammation

While inflammation is the body’s response towards injury as a healing process, uncontrolled and chronic inflammation leads to pathological conditions. Chronic inflammation plays detrimental roles in many heart and circulatory pathologies, especially in the development and progression of atherosclerosis that could lead to other CVDs including myocardial infarction, hypertension, and hypertrophic heart failure [36,37]. Cardiac inflammation is regulated through a complex interplay of enzymes, hormones, immune cells, cytokines, and receptors [38]. Although multiple factors contribute to this process, the extent of inflammation is typically assessed by the presence of immune cell infiltration at the affected site and by measuring the concentrations of pro- and anti-inflammatory cytokines in the tissue or systemic circulation [39]. Typically, cardiovascular inflammation is often associated with an increase in the levels of inflammatory cytokines such as interleukin (IL)-1β, IL-6 and tumor necrosis factor alpha (TNF-α) alongside reduced levels of anti-inflammatory cytokines including IL-10 and transforming growth factor beta (TGF-β) [40].

Although immune cells are the key drivers of cardiovascular inflammation, cardiac cells such as cardiomyocytes, fibroblasts, and endothelial cells also contribute to cytokine production in response to ischemic and hypertrophic stress [41]. Following myocardial infarction, fibroblasts undergo phenotypic modulation into alpha-smooth muscle actin (α-SMA)-expressing myofibroblasts [42]. In this activated state, they function as a dynamic signaling hub; they are highly responsive to pro-inflammatory cytokines such as tumor necrosis factor-alpha (TNF-α), IL-6, and IL-1β, while simultaneously synthesizing their own cytokines and extracellular matrix components to drive scar formation and left ventricular remodeling [43]. Furthermore, in the injured heart, resident cells utilize transcription factors such as activator protein-1 (AP-1) and nuclear factor-κB (NF-κB) to drive distinct inflammatory profiles [44]. While both cardiomyocytes and fibroblasts generate TNF-α and IL-6 in response to stress [45], IL-1β production is predominantly localized to fibroblasts, endothelial cells, and macrophages [46]. Crucially, these cells amplify inflammation through paracrine crosstalk, in which macrophage- and fibroblast-derived IL-1β prompts cardiomyocytes to release IL-6, which subsequently recruits additional leukocytes [47]. Similarly, angiotensin II relies on cardiomyocyte paracrine signaling to stimulate cytokine release from fibroblasts during fibrotic remodeling [48].

The potent paracrine effects of IL-1β in cardiovascular remodeling hinge entirely on its intracellular maturation, a process tightly governed by oxidative stress and thiol redox signaling [49]. Under conditions of oxidative stress, ROS oxidize critical reactive cysteine residues on signaling intermediates, activating the NF-κB pathway and triggering the assembly of the NOD-like receptor family pyrin domain-containing 3 (NLRP3) inflammasome [50]. The formation of the NLRP3 complex occurs in two distinct stages: priming and activation. Priming signals induce the transcription of NLRP3 components, whereas secondary triggering signals promote inflammasome assembly. This assembly leads to caspase-1 activation, the proteolytic maturation of pro-IL-1β and pro-IL-18 into their active forms, and the cleavage of gasdermin D (GSDMD), which forms membrane pores that facilitate cytokine release [51,52].

Clinically and experimentally, this oxidative NLRP3 cascade is central to diverse cardiovascular pathologies. Indeed, Zheng et al. [53] demonstrated that NLRP3 plays a critical role in atherogenesis, showing that genetic deletion of Nlrp3 in a mouse model significantly reduced atherogenic lesion formation. Similarly, pharmacological inhibition of the NLRP3 inflammasome attenuates pericardial inflammation in models of acute pericarditis, where pericardial injury releases pro-IL-1α to trigger inflammasome activation IL-1β, release, and subsequent “IL-6” -mediated inflammation. Finally, because hypertensive patients consistently exhibit elevated serum IL-1β levels, systemic NLRP3 inflammasome involvement is heavily implicated in driving the vascular inflammation that accelerates the progression of hypertension [54].

### 2.3. Dyslipidemia/Lipid Metabolism Dysregulation

Changes in the lipid profile are the primary contributors to CVD. Beyond cholesterol content, the inflammatory burden of CVD causes modifications in the lipid or protein components of high-density lipoproteins (HDL), leading to the production of defective HDL particles [55]. Reduced HDL levels impair cholesterol clearance, while elevated low-density lipoprotein (LDL) and oxidized LDL (ox-LDL) levels contribute to plaque formation in arteries. Under pathological conditions, HDL may lose its protective properties and become pro-oxidative, thereby further contributing to LDL oxidation. Moreover, HDL isolated from patients with established coronary heart disease shows a reduced ability to stimulate endothelial nitric oxide production, leading to impaired endothelial function [56]. Lipid oxidation triggers a vicious cycle of inflammation, activating inflammatory pathways that damage the endothelium and lead to atherosclerosis progression [57]. Additionally, insulin resistance and obesity contribute to hepatic lipogenesis by increasing circulating triglycerides and free fatty acids, further exacerbating lipid dysregulation [58]. Addressing lipid dysregulation through lifestyle, dietary interventions and pharmacological approaches, such as statins and PCSK9 inhibitors, is essential for reducing lipid abnormalities, mitigating oxidative stress, and ultimately reducing CVD risk.

### 2.4. Endothelial and Vascular Dysfunction

Under physiological conditions, the endothelium maintains vascular homeostasis by favouring vasodilation over constriction. It responds dynamically to intrinsic physical stimuli, with endothelial-dependent vasodilation being primarily regulated by shear stress-induced release of NO, which activates cyclic guanosine monophosphate (cGMP) production [59,60]. When NO-mediated vasodilation is impaired, compensatory mechanisms involve vasodilatory mediators such as cytochrome-derived factors, natriuretic peptides, and prostacyclin [61]. Endothelial dysfunction is often regarded as the hallmark of CVDs, characterized by impaired nitric oxide (NO) bioavailability and vascular tone regulation [62]. This dysfunction promotes vasoconstriction, platelet aggregation and vascular remodeling, creating a pro-atherogenic environment within the vessels. In states of endothelial dysfunction, the vascular environment becomes conducive to platelet and leukocyte activation and adhesion, accompanied by cytokine release that increases vessel wall permeability to oxidized lipoproteins and inflammatory mediators [63]. These processes ultimately promote structural damage, smooth muscle cell proliferation, and atherosclerotic plaque formation [64].

Endothelial NO synthase (eNOS) plays a critical role in maintaining vascular homeostasis by catalyzing the production of NO. Hence, downregulation of eNOS impairs NO bioavailability, redirecting cardiac substrate utilization from free fatty acids to lactate and thereby reducing exercise tolerance and promoting endothelial cell apoptosis [65]. Simultaneously, dysfunction of matrix metalloproteinases (MMPs) disrupts extracellular matrix remodeling, leading to pathological accumulation of structural proteins. This contributes to cardiac hypertrophy, interstitial fibrosis, and increased myocardial stiffness, culminating in progressive diastolic dysfunction [66]. Moreover, altered MMP activity compromises atherosclerotic plaque stability, heightening the risk of ischemic cardiovascular events [67].

In addition to NO, hydrogen sulfide (H_2_S) is a critical gaseous signaling molecule involved in various vascular cellular processes, including inflammation, apoptosis, cell cycle regulation, cytoprotection, and mitochondrial function [68]. Within the vasculature, H_2_S modulates vascular tone, inhibits cellular proliferation, and exerts bidirectional effects on apoptosis and autophagy in vascular smooth muscle cells (VSMCs) [69]. Impaired H_2_S signaling has been implicated in several vascular remodeling-associated pathologies, notably hypertension and atherosclerosis [69,70,71]. In hypertensive patients, diminished H_2_S biosynthesis and reduced H2S-mediated vasodilation contribute to microvascular dysfunction [72]. Lower expression levels of cystathionine γ-lyase (CSE) and cystathionine β-synthase (CBS) (both are key enzymes in H_2_S production) further suggest a dysregulated H_2_S-generating pathway in hypertension [73]. Consistently, spontaneous hypertensive rat models exhibit reduced endogenous H_2_S levels in the aorta [74]. Pharmacological inhibition of CSE using DL-propargylglycine significantly elevates basal blood pressure and promotes vascular remodeling, underscoring the essential role of H_2_S in maintaining vascular homeostasis and blood pressure regulation [75,76].

### 2.5. Cardioprotection Strategies: Translating Pathophysiology into Therapeutic Targets

Given the devastating impact of these multifactorial pathophysiological mechanisms—particularly the mitochondrial ROS burst during I/R injury—developing effective interventions is a critical therapeutic priority [31,44]. This effort is centered around the concept of cardioprotection, which encompasses all strategies and interventions designed to preserve the myocardium by minimizing cell death, reducing infarct size, and preventing adverse ventricular remodeling following an ischemic event [77,78]. In chronic cardiovascular conditions such as atherosclerosis, hypertension and progressive heart failure, cardioprotective strategies often focus on mitigating systemic lipid dysregulation, managing cardiac function, restoring nitric oxide bioavailability, and suppressing pro-fibrotic inflammatory cascades in order to suppress disease progression. In contrast, cardioprotection in acute cardiovascular events often focuses on the context of myocardial ischemia–reperfusion injury, regardless of the etiologies.

Clinically and experimentally, cardiac endogenous defenses against ischemia–reperfusion injury are activated through specific conditioning protocols that utilize transient, non-lethal ischemic episodes to trigger survival cascades [31]. These methods include ischemic preconditioning (IPC), ischemic postconditioning (IPostC), and remote ischemic conditioning (RIC). Specifically, IPC applies brief cycles of ischemia prior to a prolonged ischemic event; IPostC utilizes intermittent interruptions of blood flow at the onset of reperfusion; and RIC applies transient ischemia to a distant vascular bed. Together, these methods aim to control the pathological bursts of mitochondrial ROS, prevent calcium overload, and activate neural reflexes that protect the heart during ischemic events.

At the molecular level, mitochondria are the main mediators of cardiomyocyte survival during these protocols. The ultimate executioner of reperfusion injury is known to be the opening of the mitochondrial permeability transition pore (mPTP) [79]. These conditioning protocols prevent this by restoring the mitochondrial redox balance and activating the Reperfusion Injury Salvage Kinase (RISK) and Survivor Activating Factor Enhancement (SAFE) pathways, which inhibit pro-apoptotic proteins and seal the mPTP [45,80].

While mechanical conditioning protocols are highly effective experimentally, their clinical application during unpredictable events like acute myocardial infarction remains challenging. Consequently, research on acute cardiovascular event management has shifted toward identifying pharmacological mimetics, which are compounds that are capable of chemically infiltrating the mitochondria to scavenge pathological ROS, inhibit mPTP opening, and activate survival kinases without the need for mechanical ischemia [80,81]. As maintenance of the intracellular GSH pool and H_2_S signaling are central to these mitochondrial defenses, exogenous sulfur-donating compounds, particularly cysteine and its derivatives, have emerged as highly promising cardioprotective candidates.

## 3. Cysteine

**Figure 1:**
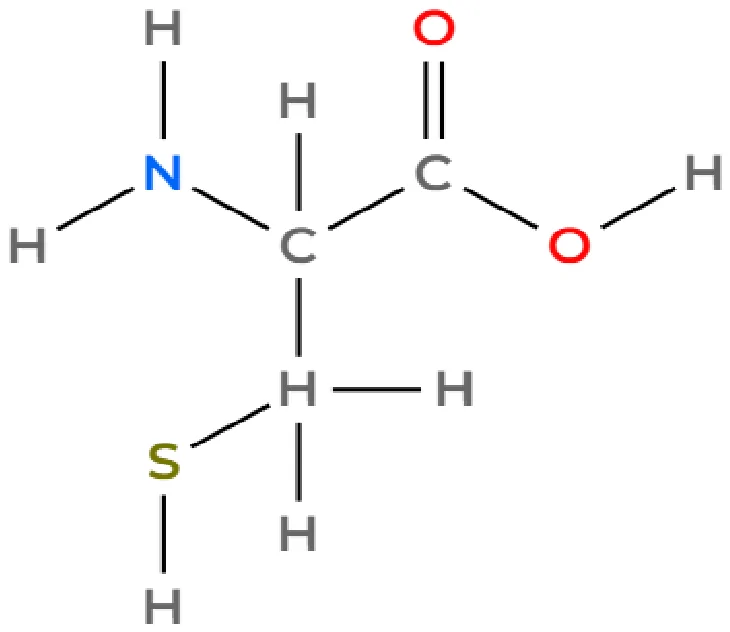
Structure of cysteine (C_3_H_7_NO_2_S).

**Figure 2:**
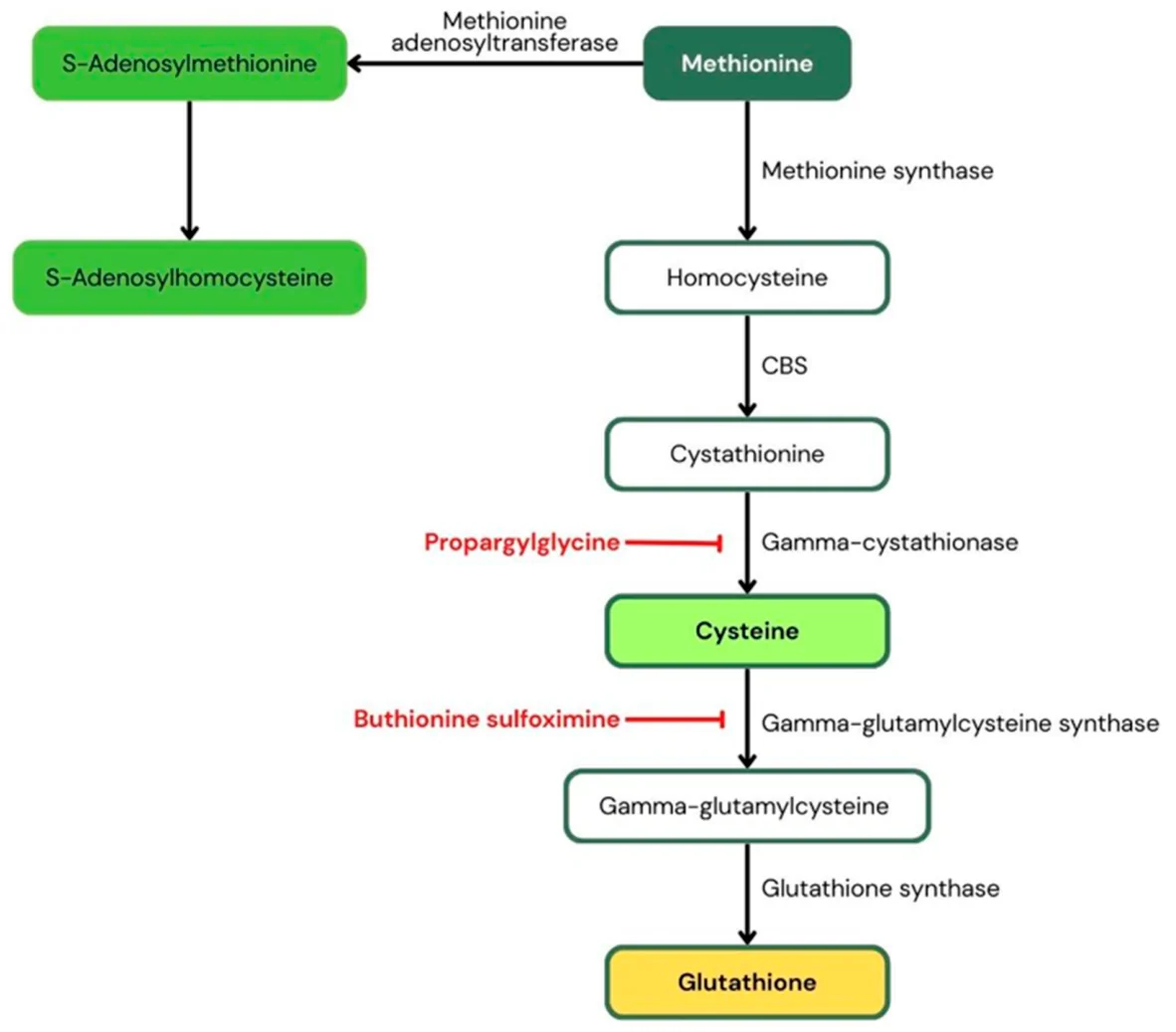
The trans-sulfuration pathway connects methionine and glutathione biosynthesis. In the methionine cycle, methionine forms S-adenosylmethionine which serves as a methyl donor, generating S-adenosylhomocysteine. This is converted to homocysteine, which is subsequently converted back into methionine. Homocysteine has an alternative fate, however. It can be used to produce cystathionine, which is further converted to cysteine. This latter conversion is catalyzed by gamma-cystathionase and inhibited by propargylglycine. Cysteine can then feed glutathione biosynthesis through production of gamma-glutamylcysteine. This step is catalyzed by gamma-glutamylcysteine synthase and inhibited by buthionine sulfoximine.

Structurally, cysteine comprises a carboxylic group (COOH), amino acid group (NH_2_), and thiol group (-SH) (Figure 1). The chemical formula of cysteine is C_3_H_7_NO_2_S, and it has a molecular weight of approximately 121.16 g/mol. Its three-dimensional structure highlights the thiol group’s ability to form disulfide bonds, which are critical for stabilizing protein structures and facilitate redox reactions that neutralize ROS. Common dietary sources of cysteine include eggs, chicken, garlic, and broccoli. In the liver, cysteine is synthesized from methionine via the trans-sulfuration pathway, which involves a series of biochemical enzymes including cystathionine γ-lyase (CSE) and cystathionine β-synthase (CBS) (Figure 2) [82,83]. The presence of the -SH group and its ability to donate hydrogen atoms contribute to its free radical scavenging and ROS-neutralizing properties, making cysteine an important dietary-sourced antioxidant [84]. Table 1 shows cysteine derivatives and their protective effects in preclinical studies of various diseases.

The trans-sulfuration pathway refers to the metabolic process that converts homocysteine to cysteine, primarily involving CBS and CSE (Figure 2) [92]. This pathway involves the transfer of sulfur atoms from methionine to serine, yielding cysteine as a product. The first step to the trans-sulfuration pathway is the formation of S-adenosylmethionine from the activation of methionine that reacts with adenosine triphosphate (ATP) [93]. S-adenosylmethionine is a key methyl donor, playing an important role in the synthesis of creatine and several neurotransmitters [94]. The transfer of a methyl group from S-adenosylmethionine produces S-adenosylhomocysteine. In physiological conditions, the tissue level of S-adenosylhomocyteine is generally low because this compound is rapidly cleaved into homocysteine and adenosine by a specific hydrolase [95]. Following its rapid cleavage, about half of the homocysteine generated is re-methylated into methionine, while the rest are condensed with serine by CBS to form cystathionine [96]. The cleavage of cystathionine is catalyzed by enzyme CSE or also known as cystathionase that functions almost entirely to produce cysteine [97]. The cysteine produced then can be used in the glutathione biosynthesis via production of glutamate-cysteine ligase (also known as gamma-glutamylcysteine), catalyzed by gamma-glutamylcysteine synthase [98].

### 3.1. Cysteine-Containing Compounds and Cardiovascular Benefits

**Figure 3:**
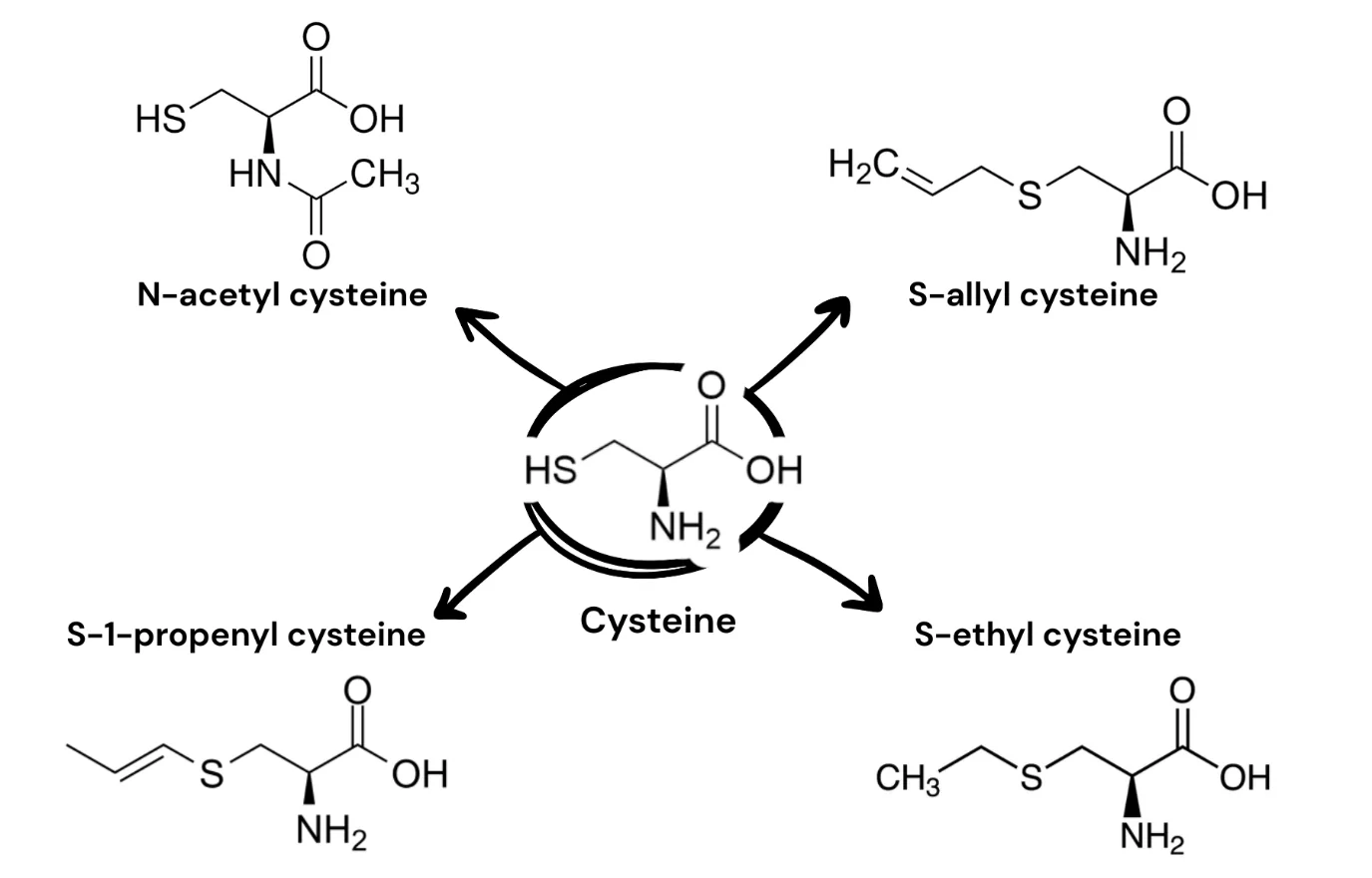
Derivatives of cysteine.

Glutathione (GSH) is a tripeptide of glutamate, glycine, and cysteine, with the cysteine thiol group serving as its active site. This thiol group enables GSH to act as a major intercellular antioxidant. In the cardiovascular system, glutathione is a key antioxidant that maintains redox homeostasis, detoxifies ROS, and protects myocardial and endothelial function, particularly in aging hearts [99,100]. Consequently, low GSH levels are linked to hypertension and heart failure [101]. While glutathione depletion naturally occurs through oxidation to glutathione disulphide (GSSG) or conjugation with xenobiotics, its poor bioavailability severely limits its direct therapeutic supplementation. Instead, boosting intracellular glutathione through cysteine-containing compounds such as N-acetyl cysteine, S-allyl cysteine, S-propenyl cysteine, and S-ethyl cysteine (Figure 3) has become a primary focus of current research.

**Figure 4:**
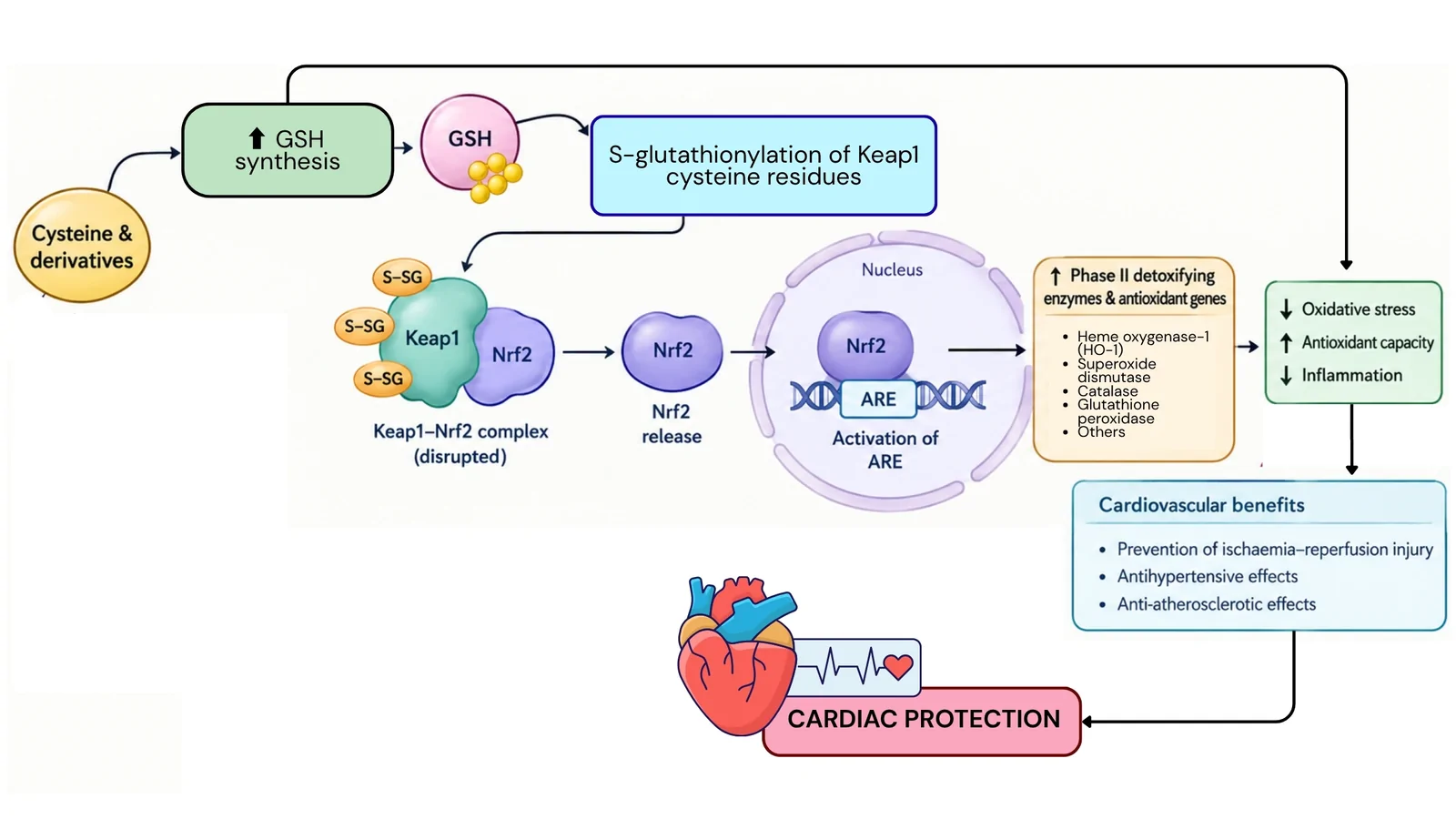
Schematic representation of cysteine’s role in cardiovascular protection via the Nrf2 pathway.

In preclinical models, these cysteine derivatives offer cardiovascular protection not only by providing the rate-limiting building blocks to enhance glutathione levels but also by facilitating the activation of the nuclear factor erythroid 2-related factor 2 (Nrf2) pathway to mitigate oxidative stress [102,103]. A crucial mechanism underlying these protective effects involves Nrf2 activation via S-glutathionylation, which disrupts the Kelch-like ECH-associated protein 1 (Keap1)-Nrf2 complex [104]. The Nrf2 transcription factor, essential for cellular defense against oxidative stress, is primarily regulated by its interaction with the Keap1 protein, which contains critical cysteine residues [105]. Cysteine compounds directly interact with these residues, which are crucial for sensing oxidants [106]. When these specific Keap1 cysteines undergo modifications such as oxidation, S-glutathionylation, orand adduction by electrophiles, Nrf2 is released from the Keap1-Nrf2 complex. This release allows Nrf2 to translocate to the nucleus and subsequently activate the transcription of phase II detoxifying enzymes (Figure 4) [105,107].

However, direct Keap1 modification is not equally established for all cysteine derivatives. For instance, S-1-propenyl cysteine (S1PC) activates the Nrf2 predominantly through an indirect mechanism, which is by promoting the degradation of BTB domain and CNC homolog 1 (BACH1), rather than via modification of Keap1. BACH1 is a known transcriptional repressor of Nrf2 [91,108]. Hence, while utilizing cysteine-containing compounds offers a highly promising therapeutic strategy for protecting cardiovascular via Nrf2 axis, their distinct upstream molecular targets must be carefully delineated.

#### 3.1.1. N-Acetyl Cysteine

N-acetyl cysteine (NAC) is a synthetic derivative of L-cysteine and a crucial precursor to GSH. The therapeutic properties of NAC have been widely explored through both in vitro and in vivo research, supporting its clinical use for more than five decades. Its initial clinical value as a mucolytic agent was reported in the 1960s in individuals with cystic fibrosis. By the 1970s, NAC became the established treatment for acetaminophen (paracetamol) poisoning. Since the 1980s, beyond its approved roles as a mucolytic and an antidote for acetaminophen toxicity, its potential utility has been examined in numerous disorders in which oxidative stress is considered a central contributor to disease development and progression [109,110].

Glutathione is synthesized in two steps from cysteine, glutamate and glycine, with cysteine being the rate-limiting factor due to its low intracellular concentration during oxidative stress [111]. NAC serves as a more stable and efficient source of antioxidant sulfuhydryl moieties than L-cysteine, which is rapidly oxidized to its disulphide form in solution [112]. NAC easily crosses the plasma membrane’s phospholipid bilayer. Once inside the cell, it is rapidly hydrolysed to cysteine for GSH production [113]. While NAC primarily acts in vivo as an indirect antioxidant via this GSH precursor pathway, its nucleophilic free sulfhydryl group also acts directly against electrophilic oxidant radicals, such as protecting a1-antitrypsin from inactivation by hypochlorous acid [114].

Clinically, NAC has been investigated as an adjunct therapy with which to treat cardiac ischemia–reperfusion damage, stable angina pectoris, cardiomyopathy, and cardiotoxicity brought on by doxorubicin treatment. In patients with coronary bypass surgery grafts, NAC attenuates inflammation and the incidence of postoperative atrial fibrillation [115,116]. However, its broader cardiovascular mechanisms are primarily understood through experimental models. For example, NAC treatment attenuated particulate matter (PM)-potentiated atherosclerosis by alleviating the excessive plasma ROS levels, substantially decreasing the levels of circulating oxidized LDL (ox-LDL), and restoring endothelial progenitor cells (EPCs), thereby mitigating inflammatory cytokines in LDLR-knockout mice fed a high-fat diet [117]. Another animal study by Fang et al. [89] revealed that NAC treatment elevated the levels of superoxide dismutase-1 (SOD-1) and glutathione peroxidase-1 (GPX-1), thereby alleviating methylglyoxal (MG)-dicarbonyl stress and oxidative stress in diabetic Apo-E knockout mice. Furthermore, the study also indicated the restoration of endothelial damage, as NAC increased the expression levels of phosphorylated (p-)Akt and p-eNOS in the aorta, associated with increased nitric oxide in serum. Additionally, NAC treatment inhibited excessive autophagy activity, as observed in the ratio of the autophagic markers LC3 II/I, p62, AMPK, and mTOR in male diabetic rats with heart ischemia–reperfusion injury [79].

Regarding its pharmacokinetic and safety profile, the oral bioavailability of intact NAC is relatively low (approximately 6–10%) due to rapid and extensive first-pass hepatic metabolism, where it is quickly deacetylated into L-cysteine and glutathione [109]. Consequently, while oral doses (typically 600–1200 mg daily) are common for chronic antioxidant support, intravenous (IV) administration is frequently utilized in acute settings to rapidly achieve therapeutic plasma concentrations [118,119]. While generally safe, high-dose IV NAC can induce adverse effects, primarily gastrointestinal distress, flushing, and dose-dependent non-immunologic anaphylactoid reactions [120].

#### 3.1.2. S-Allyl Cysteine

SAC is a garlic-derived organosulfur compound in the hydrophilic fraction of aged garlic extract (AGE) that is associated with cardioprotective effects [121,122]. It has been identified as the most prevalent and active organosulfur compound in AGE [123]. Structurally derived from L-cysteine, it incorporates an allyl moiety and thioether sulfur, which confers its unique pharmacokinetic and functional properties [124]. SAC is notable for its chemical stability, favorable safety profile, and broad spectrum of biological effects [125]. It exhibits high oral bioavailability and long systemic persistence; in rodents, pharmacokinetic studies have documented nearly complete oral absorption, limited metabolism, and extensive renal absorption [126,127]. Its stability is partly attributed to its C-S bond cleavage under acidic conditions, which facilitates rapid gastrointestinal absorption after oral administration [80]. A recent comprehensive review confirms that SAC circulates largely in its intact form in plasma [128], demonstrating prolonged systemic retention due to its relatively low susceptibility to N-acetylation [126]. In a human study, plasma SAC levels remained detectable up to 24 h post-administration [80].

Beyond its favorable pharmacokinetic profile, SAC is a potent modulator of cellular redox homeostasis, functioning primarily through the activation of the Nrf2 signaling pathway [123]. As a thiol-containing compound, SAC promotes the dissociation of Keap1 from through direct cysteine modification, preventing the degradation of Nrf2 and enabling its protective effect [129], leading to the upregulation of endogenous cytoprotective enzymes such as heme oxygenase-1 (HO-1), SOD, catalase, and GPX. Through these mechanisms, SAC has shown promise in animal models for mitigating ischemia/reperfusion injury by upregulating endogenous antioxidants, preserving mitochondrial membrane potential, and reducing lactate hydrogenase leakage in the myocardium [81].

In addition to its role in redox-sensitive transcriptional regulation, SAC’s sulfur containing structure has potential involvement in sulfur-based post-translational modifications, particularly S-sulfhydration (persulfidation) [130]. This modification is crucial for the biological effects of hydrogen H_2_S, including antioxidant, anti-inflammatory, and vasorelaxation properties, as well as pro-angiogenesis and mitigation of myocardial ischemia–reperfusion [131,132]. Although in vivo evidence of SAC-induced protein S-sulfhydration remains limited, in vitro studies demonstrate that SAC stimulates H_2_S release in endothelial cells, improving endothelial health by attenuating ROS and increasing eNOS [88]. Another pathway SAC uses to stimulate H_2_S production is through the endogenous enzyme CSE, which is predominantly found in the myocardium and vascular smooth muscle cells [133].

Preclinical evidence indicates that SAC exerts cardioprotective effects that extend to sustaining endothelial function and modulating vascular tone. One of the primary mechanisms involves the modulation of NO bioavailability [134]. SAC activates the Akt/endothelial nitric oxide synthase signaling pathway, stimulating endothelial progenitor cells (EPCs) to enhance neurovasculogenesis and blood flow recovery in ischemic tissues [135]. Prior evidence suggests that Nrf2 activation can also influence eNOS expression and NO production [136,137]. Furthermore, SAC and related derivatives have been implicated in the downstream modulation of NO-cGMP pathways [138]. In a randomized clinical trial, the blood pressure of individuals with Grade I hypertension undergoing antihypertensive drug therapy was significantly reduced by low doses of SAC (about 0.25 mg per day) [139]. A mechanistic study by Syu et al. [135] demonstrated that SAC stimulates the Akt/endothelial nitric oxide synthase signaling pathway, promoting neovasculogenesis and vascular repair, resulting in improved blood flow recovery in ischemic tissues. However, the magnitude of this effect and its clinical relevance remain insufficiently defined and warrant more targeted investigation.

SAC is frequently associated with antithrombotic effects, complementing its lipid and blood pressure modulating actions. While acute inhibition of platelet aggregation IIb/IIIa receptors is more consistently attributed to highly reducing oxidative stress in circulating platelets, preserving membrane fluidity, and maintaining receptor functionality under elevated ROS [78,140]. SAC’s capacity to strengthen endogenous antioxidant systems and limit pathological ROS accumulation supports the attenuation of maladaptive cardiac remodeling and the reduction of fibrotic progression [141,142].

From a clinical safety perspective, SAC and standardized aged garlic extracts demonstrate an exceptional safety profile, with clinically achievable doses (such as roughly 0.25 mg/day of SAC) administered in human trials with negligible adverse events [139]. However, drug interactions with standard cardiovascular medications warrant careful consideration. Preclinical studies reveal that SAC exhibits super-additive, synergistic blood pressure-lowering effects when combined with ACE inhibitors like captopril [87] or β-blockers like carvedilol [87]. While this synergy is beneficial for restoring vascular tone, it necessitates the dose titration of primary antihypertensives to avoid hypotensive episodes. Additionally, because SAC and garlic extracts preserve platelet membrane fluidity and modulate aggregation [78,121], concurrent administration with standard antithrombotic agents or oral anticoagulants (e.g., warfarin) may theoretically heighten cumulative bleeding risks, warranting caution in patients undergoing antiplatelet therapy [143].

#### 3.1.3. S-1-Propenyl Cysteine

While SAC demonstrates extended blood retention due to its low susceptibility to acetylation, it is not the sole bioactive organosulfur mediator in AGE. Discovered in the 1960s, S-1-propenyl cysteine (S1PC), which is a stereoisomer of SAC, has emerged in preclinical studies as a distinct, independent driver of cardiovascular and immunomodulatory protection [144,145,146]. Notably, in hypertensive animal models, S1PC administration significantly attenuates systolic blood pressure by reducing peripheral vascular resistance, which is a hallmark pathology of hypertension [147,148]. Importantly, this antihypertensive effect is not mirrored by other AGE derivatives, including SAC, highlighting the unique pharmacological profile of the S1PC stereoisomer [148].

The mechanisms underlying this vascular improvement rely heavily on NO signaling pathways. Under stress conditions, S1PC elevates circulating nitrogen oxide metabolites and promotes the phosphorylation of both AMP-activated protein kinase (AMPK) and eNOS in the aortic wall, establishing an active AMPK/eNOS/NO signaling axis [149]. Serum NO serves as a critical indicator of NO homeostasis, which is essential for vasodilation, platelet inhibition, and vascular tone regulation; consequently, impaired NO bioavailability is deeply implicated in the pathogenesis of hypertension and atherosclerosis [150,151,152].

Crucially, this S1PC-induced NO production intersects directly with cellular antioxidant defenses. In the presence of NO donors, S1PC promotes the degradation of BTB domain and CNC homolog 1 (BACH1), a transcriptional repressor of Nrf2 [91,108]. By removing this repressor, S1PC facilitates the nuclear translocation of Nrf2 and the subsequent upregulation of antioxidant response element (ARE)-driven genes, including heme oxygenase-1 (HO-1), thereby conferring robust endothelial cytoprotection against oxidative injury [108].

Beyond vascular tone and redox balance, S1PC exerts profound anti-inflammatory effects that mitigate chronic atherosclerosis. It has been shown to activate autophagy, which subsequently suppresses Toll-like receptor 4 (TLR) signaling, a primary driver of pro-atherogenic vascular inflammation [152,153]. Furthermore, S1PC actively reshapes the immune microenvironment by boosting IL-10-induced arginase-1 (Arg1) expression and driving macrophage polarization toward the anti-inflammatory M2c phenotype, while simultaneously suppressing pro-inflammatory M1-like markers and lipopolysaccharide (LPS)-induced cytokine production [154]. While S1PC clearly delivers multifaceted cardioprotection, the precise extent to which Nrf2 activation mediates these specific downstream anti-inflammatory and immunomodulatory effects requires further elucidation.

Despite these robust preclinical mechanisms, the clinical utility of S1PC is currently limited by a lack of comprehensive human pharmacokinetic data. While animal models demonstrate its rapid systemic absorption and distinct pharmacological behavior compared to SAC [128,148], specific parameters regarding its human bioavailability, clinically achievable plasma concentrations, and potential interactions with standard antihypertensive or antiplatelet medications remain undefined and require dedicated clinical evaluation.

#### 3.1.4. S-Ethyl Cysteine

S-ethyl cysteine (SEC) is a water-soluble alkylated derivative of cysteine in which the thiol hydrogen group is substituted by an ethyl group [155,156]. This compound occurs naturally as one of the several S-alk(en)-L-cysteine derivatives found in garlic and aged garlic preparations, although it remains considerably less studied than SAC and S1PC. Like other garlic-derived cysteine conjugates, SEC has been implicated as a bioactive constituent that contributes to the antioxidant and organ protective properties. SEC is structurally related to other S-alkylated cysteine compounds such as SAC and S-methyl-L-cysteine, which have been more extensively studied for their antioxidant and cardioprotective effects. However, evidence specifically addressing the cardiovascular effects of SEC remains limited. To date, only one in vivo rodent study has directly examined SEC in a cardiometabolic context, reporting that SEC ameliorated high-fat diet-induced metabolic disturbance, oxidative stress, inflammation, and dyslipidemia in rodents, factors closely linked to the development and progression of cardiovascular disease [157]. Although these findings provide preliminary evidence of potential cardiovascular relevance, they do not establish direct cardioprotective effects of SEC.

Evidence from non-cardiac models may provide further mechanistic insights. SEC has demonstrated protective effects in other organ systems, particularly the nervous system and kidneys, where it has been associated with reductions in oxidative stress, inflammation, and mitochondrial dysfunction [158,159]. These processes are also implicated in cardiovascular pathologies, including myocardial ischemia–reperfusion injury, endothelial dysfunction, and metabolic cardiomyopathy. However, the presence of shared pathological pathways does not necessarily indicate that SEC produces equivalent protective effects in cardiovascular tissues. Thus, findings from renal and other organ systems should be considered as mechanistic evidence that may support, rather than establish, a potential cardiovascular protective role for SEC.

Similarly to S1PC, the pharmacokinetic profile and safety considerations of SEC are poorly characterized. Because current cardiovascular-related evidence relies heavily on high-fat diet rodent models [157], critical translational parameters, including the optimal route of administration, human metabolism, therapeutic dosage, and potential adverse interactions with lipid-lowering drugs (e.g., statins), remain completely unknown. Establishing these pharmacokinetic baselines is a mandatory prerequisite before SEC can be evaluated for clinical cardiovascular therapies.

Taken together, the current literature supports the hypothesis that SEC may exert cardiovascular benefits through modulation of redox homeostasis, inflammatory signaling, and metabolic dysfunction. Nevertheless, direct evidence demonstrating cardioprotective effects remains scarce, and the cardiovascular activity of SEC should therefore be regarded as a hypothesis requiring further validation. Future studies using cardiovascular-specific models are needed to determine whether the effects observed in other organ systems translate to cardiac and vascular tissues and to define the molecular targets, dose–response relationships, and therapeutic relevance of SEC in cardiovascular disease.

## 4. Future Therapeutic Potential of Cysteine-Containing Natural Compounds in Cardiovascular Disease Management

While preclinical data provide robust mechanistic insights into the cardioprotective effects of cysteine and its derivatives, translating these findings requires rigorous evaluation in human subjects. Presently, clinical evidence remains limited but still very much promising.

As previously discussed, researchers worldwide recommend the inclusion of specific nutraceuticals, such as cysteine-containing products, as an adjunct therapy to conventional treatments for CVDs [160,161]. These products may enhance therapeutic efficacy by providing additional antioxidant protection and have the potential to transform the landscape of cardiovascular disease treatment. Human studies have primarily investigated NAC in surgical settings and SAC-containing garlic extracts in hypertensive patients. However, to fully establish their therapeutic efficacy, these studies must be critically evaluated in terms of their sample sizes, treatment duration, and specific clinical endpoints. Table 2 summarizes the key clinical trials evaluating cysteine-based compounds in cardiovascular health.

In addition, several countries have registered products that contain cysteine as their main ingredient for clinical trials that are currently ongoing. They aim to use these products as dietary interventions to reduce the risk of cardiovascular conditions such as hypercholesterolemia, hypertension and hyperlipidemia, among hundreds of others [162,163,164]. Furthermore, proprietary cysteine-containing products have been formulated to combine dietary sources of NO and H_2_S to increase the levels and duration of NO biosynthesis; these interventions aim to ameliorate high blood pressure in hypertensive patients [164]. However, it is critical to distinguish trial registration from demonstrated clinical efficacy. Registered or ongoing studies cannot be considered evidence of therapeutic effectiveness until their results are fully available, rigorously peer-reviewed, and adequately evaluated. Until such data is published, these interventions remain investigational candidates rather than proven clinical therapies. Nevertheless, these clinical trial data, once available, may underpin successful nutraceutical interventions of cysteine-containing products in reducing cardiovascular risk and serving as adjunctive therapies for patients with CVDs.

**Table 2:** Summary of clinical trials evaluating cysteine-containing compounds for cardiovascular health.

Compound	Study Design and Sample Size	Treatment Duration and Administration	Experimental Groups	Primary Clinical Endpoints	Key Findings and Study Limitations	Reference
N-acetyl cysteine (NAC)	Double-blind randomized controlled trial (RCT) (*n* = 115)	Post-coronary artery bypass graft (CABG) patients, hospitalization period Treatment via intravenous	IV NAC + metoprolol vs. Placebo + metoprolol	Incidence of postoperative atrial fibrillation (POAF)	Findings: Significant reduction in POAF incidence Limitations: Single-center design; short-term follow up; exclusively surgical context	Soleimani et al. [116]
NAC	RCT (*n* = 260)	Cardiac surgery patients (post-operative) Treatment via oral administration	Carvedilol + NAC vs. Carvedilol alone vs. metoprolol	POAF incidence; oxidative stress markers	Findings: Combination therapy synergistically reduced POAF and oxidative stress. Limitations: Did not evaluate long-term cardiovascular mortality	Ozaydin et al. [115]
S-allyl-cysteine (SAC)	Triple-blind RCT (*n* = 81)	Grade I hypertension Treatment via oral administration for 12 weeks	Optimized aged garlic extract (0.25 mg/day) vs. Placebo	Systolic and diastolic blood pressure	Findings: Significant blood pressure reduction in patients on standard therapy. Limitations: Small sample size; unmeasured dietary sulfur intake during trial	Serrano et al. [139]
Glutathione (GSH)	RCT (*n* = 54)	Healthy adults Treatment via oral administration	GSH (250 or 1000 mg/day) vs. Placebo	Systemic redox status; erythrocyte GSH levels	Findings: Elevated erythrocytes GSH pool by 30–35%. Limitations: Healthy cohort without established CVD; indirect cardiovascular implications	Richie et al. [165]
GSH	RCT	Postmenopausal women Treatment via oral administration	GSH + Citrulline vs. Placebo	Endothelial function; blood pressure; endothelial reactivity	Findings: Improved endothelial function and blood pressure Limitations: Co-administered with citrulline, making isolated GSH effects harder to distinguish	Figueroa et al. [99]
Garlic (source of SAC)	RCT, double-blind	Patients with risk of ischemic heart attack Treatment via oral administration	Garlic vs. Control	Platelet aggregation	Findings: Decreased platelet aggregation. Limitations: Older study; evaluated whole garlic rather than isolated SAC	Kiesewetter et al. [121]

**Figure 5:**
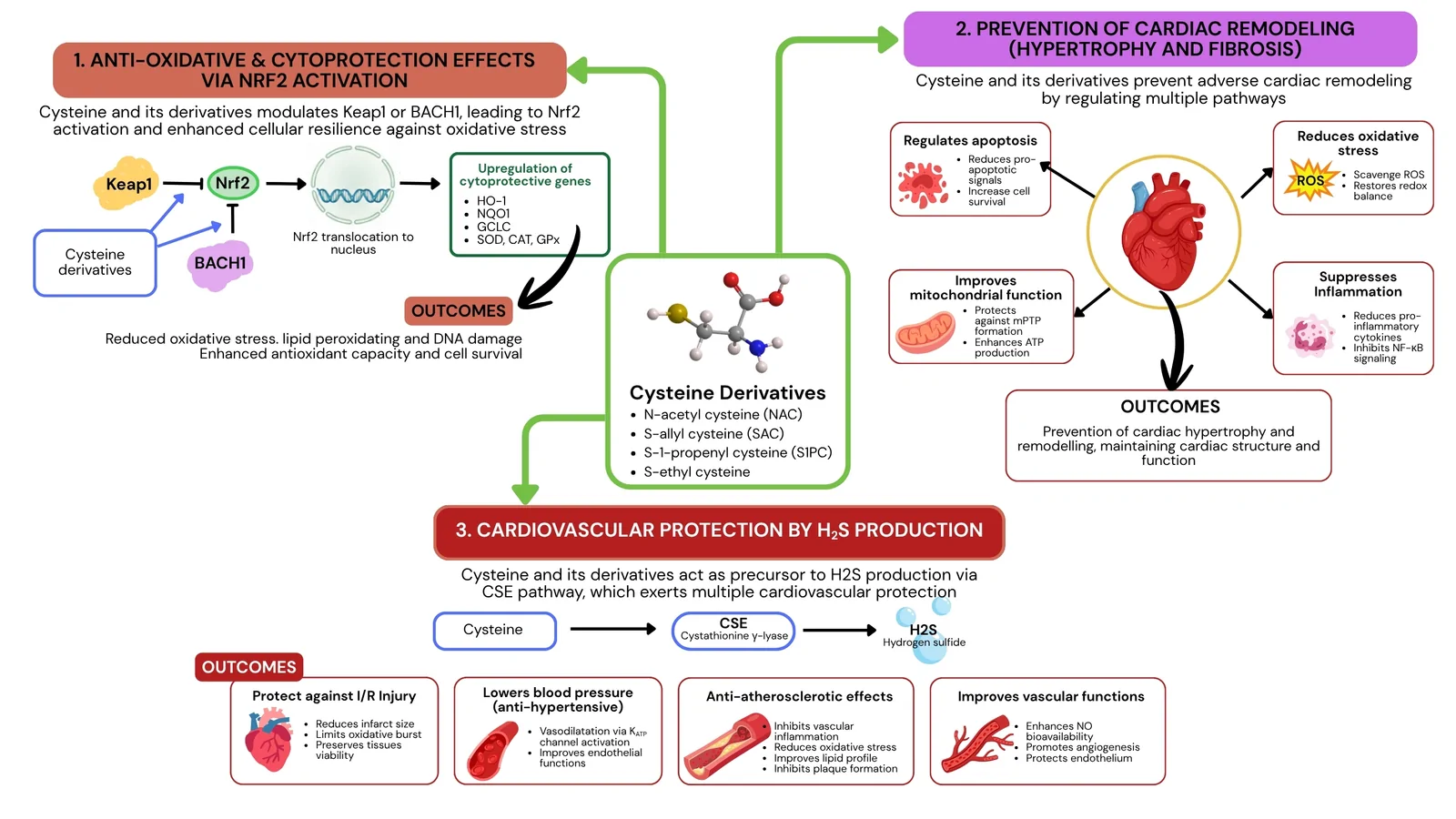
Summary of the cardiovascular protective properties of cysteine derivatives.

To conclude, as summarized in Figure 5, studies have shown that cysteine and its derivatives play a highly promising role in cardiovascular defense by modulating oxidative stress, inflammation, and mitochondrial function. Increasing evidence from both in vivo and in vitro studies demonstrates their experimental cardioprotective potential. These compounds exhibit beneficial effects by targeting pathways such as Nrf2, thereby enhancing cellular resilience against oxidative damage. Furthermore, their role as a precursor of H_2_S production provides a strong mechanistic basis for mitigating ischemia–reperfusion injury, hypertension, and atherosclerosis in laboratory settings. Table 3 provides a clear synthesis of the current evidence linking each specific cysteine derivative to these major cardiovascular pathologies.

Despite these promising and robust preclinical findings, challenges remain in translating these benefits into clinical practice, as variations in dosage, bioavailability, and metabolism influence therapeutic efficacy. The growing interest in nutraceutical interventions highlights the need for long-term clinical trials to establish the safety and effectiveness of cysteine-containing compounds in cardiovascular disease management. While the concept of using cysteine-based nutraceuticals as adjunctive therapies is compelling, describing them as safe, effective, or transformative is premature until validated by robust human data. Future studies must prioritize standardizing extract preparations, defining optimal therapeutic dosages, and monitoring long-term safety profiles to validate these nutraceutical interventions. Ultimately, the future of cysteine in cardiovascular medicine lies in its integration with conventional pharmacological treatments. With further advancements in formulation technology and robust clinical validation, cysteine and its derivatives are poised to become a safe, effective, and transformative adjunctive strategy for combating oxidative stress-related cardiovascular disorders.

## Figures and Tables

**Table 1:** Cysteine-containing compounds and their cardioprotective effects.

Cysteine Derivative	Study Design	Dosage Information	Findings	References
S-allyl cysteine (SAC)	In vivo Male adult Wistar rats Model: acute myocardial infarction	50 mg/kg/day	CSE/H_2_S Pathway: Upregulated CSE activity and H_2_S levels; significantly reduced infarct size and improved left ventricular remodeling.	Chuah et al. [77]
SAC	In vivo Male albino Wistar rats Model: isoprenaline-induced cardiac injury	50, 100, and 150 mg/kg daily for 45 days	Membrane Stabilization: Modulated membrane-bound enzymes and exhibited antioxidant properties comparable to α-tocopherol.	Padmanabhan et al. [85]
S-allyl cysteine sulphoxide (SACS) and fresh garlic homogenate (FGH)	In vivo Female Wistar albino rats Model: hypertension	SACS: 0.11 mg/kg and 0.222 mg/kg FGH: 125 mg/kg (low dose) and 250 mg/kg (high dose)	ACE Inhibition: Synergistic reduction of systolic blood pressure when combined with captopril. Enhanced therapeutic effects were observed when garlic was used alongside captopril.	Asdaq & Inamdar [86]
Aged Garlic Extract (AGE) and S-allylcysteine	In vivo Female Sprague Dawley rats Model: isoprenaline-induced cardiac injury	AGE: 2 mL/kg and 5 mL/kg SAC: 13.1 mg/kg and 32.76 mg/kg	Antioxidant Defense: Increased SOD and catalase; decreased myocardial damage markers (LDH, CK-MB).	Asdaq et al. [87]
SAC and black garlic extract	In vitro cells Bovine Aortic Endothelial cells (BAE-1)	100 μM	eNOS/NO Pathway: Enhanced eNOS phosphorylation; stimulated direct H_2_S and NO release while reducing intracellular ROS.	Geddo et al. [88]
N-acetyl cysteine (NAC)	In vivo heart Model: diabetic + ischemic–reperfusion injury	Oral	AMPK/mTOR Pathway: Inhibited excessive autophagy activity (LC3 II/I, p62, AMPK) to prevent ischemic damage.	Wang et al. [79]
NAC	In vivo vasculature Model: diabetic Apo-E KO mice)	Oral	Akt/eNOS Axis: Upregulated SOD-1/GPX-1; alleviated dicarbonyl stress; restored p-Akt and p-eNOS.	Fang et al. [89]
S-1-propenyl cysteine (S1PC)	In vivo vasculature Model: Spontaneous hypertensive rats (SHR)	S1PC administration	AMPK/eNOS/NO Axis: Promoted phosphorylation of AMPK and eNOS in the aortic wall, lowering peripheral resistance.	Ushijima et al. [90]
S1PC	In vitro Endothelial cells	S1PC with NO donors	BACH1/Nrf2 Axis: Promoted degradation of BACH1, facilitating Nrf2 nuclear translocation and HO-1 upregulation.	Tsuneyoshi et al. [91]

**Table 3:** Synthesis of preclinical and clinical evidence linking cysteine derivatives to specific cardiovascular conditions.

Cysteine Derivative	Hypertension	Atherosclerosis and Dyslipidemia	Ischemia–Reperfusion Injury	Cardiac Remodeling
N-acetyl cysteine (NAC)	Modulates vascular tone by restoring NO bioavailability and protecting eNOS. [116]	Decreases circulating ox-LDL, restores endothelial progenitor cells, and mitigates particulate matter-induced vascular inflammation. [115]	Scavenges mitochondrial ROS and inhibits excessive autophagic flux (e.g., LC3 II/I modulation) during ischemic events. [117]	Used adjunctively for cardiomyopathy; reduces postoperative inflammation and atrial fibrillation post-cardiac surgery. [113]
S-allyl cysteine (SAC)	Reduces systolic blood pressure in human trials; stimulates Akt/eNOS pathway for vascular repair. [132]	Modulates lipid profiles and exerts antithrombotic effects by preserving platelet membrane fluidity. [118,137,138]	Preserves mitochondrial membrane potential, reduces LDH leakage, and upregulates endogenous antioxidants (SOD, GPX). [77,80]	Limits pathological ROS accumulation to attenuate maladaptive structural remodeling and reduce fibrotic progression. [78,139]
S-1-propenyl cysteine (S1PC)	Attenuates systolic blood pressure by activating the AMPK/eNOS/NO signaling axis to reduce peripheral vascular resistance. [144,145]	Promotes M2c macrophage polarization, activates autophagy to suppress TLR4 signaling, and lowers pro-atherogenic cytokines. [151,152,153]	Upregulates HO-1 via NO-dependent degradation of the BACH1 repressor, conferring endothelial cytoprotection. [149,150]	Anti-inflammatory pathways suggest potential anti-remodeling benefits, but direct structural studies are lacking.
S-ethyl cysteine (SEC)	Inferred hypotensively from its ability to mitigate systemic metabolic disturbances. [156]	Ameliorates high-fat diet-induced dyslipidemia and systemic oxidative stress in rodent models. [156]	Hypothesized to confer protection based on mitochondrial preservation observed in non-cardiac (renal/neural) models. [157,158]	Direct investigations into cardiac hypertrophy and ventricular fibrosis remain to be established.

## Data Availability

No new data were created or analyzed in this study. Data sharing is not applicable to this article.

## Abbreviations

The following abbreviations are used in this manuscript:

AGE	Aged garlic extract
AMI	Acute myocardial infarction
AMPK	AMP-activated protein kinase
AP-1	Activator protein-1
ARE	Antioxidant response element
Arg1	Arginase-1
ATP	Adenosine triphosphate
BACH1	BTB domain and CNC homolog 1
BAE-1	Bovine aortic endothelial cells
BGE	Black garlic extract
CBS	Cystathionine β-synthase
cGMP	Cyclic guanosine monophosphate
COOH	Carboxylic group
CSE	Cystathionine γ-lyase
CVD/CVDs	Cardiovascular disease/Cardiovascular diseases
eNOS	Endothelial NO synthase/Endothelial nitric oxide synthase
EPCs	Endothelial progenitor cells
FGH	Fresh garlic homogenate
GPX	Glutathione peroxidase-1
GPx4	Glutathione peroxidase 4
GSDMD	Gasdermin D
GSH	Glutathione
GSSG	Glutathione disulphide
H_2_O_2_	Hydrogen peroxide
H_2_S	Hydrogen sulfide/Hydrogen sulfide
HDL	High-density lipoproteins
HO	Heme oxygenase-1
IL	Interleukin
Keap1	Kelch-like ECH-associated protein 1
LDL	Low-density lipoprotein
LPS	Lipopolysaccharide
MG	Multidisciplinary Digital Publishing Institute
MMPs	Methylglyoxal matrix metalloproteinases
NAC	N-acetyl cysteine
NF-κB	Nuclear factor-κB
NH_2_	Amino acid group
NK	Natural killer
NLRP3	NOD-like receptor family pyrin domain-containing 3
NO	Nitric oxide
Nrf2	Nuclear factor erythroid 2-related factor 2
O_2_^−^	Superoxide
•OH	Hydroxyl radical
ox-LDL	Oxidized LDL/Oxidized low-density lipoprotein
p-Akt	Phosphorylated (p-)Akt
PAF	Platelet-activating factor
PHGP	Phospholipid hydroperoxide glutathione peroxidase
PM	Particulate matter
ROS	Reactive oxygen species
S1PC	S-1-propenyl cysteine
SAC	S-allyl cysteine/S-allylcysteine
SACS	S-allyl cysteine sulphoxide
SEC	S-ethyl cysteine
SH	Thiol/Thiol group
α-SMA	Alpha-smooth muscle actin
SOD	Superoxide dismutase
SPC	S-propyl cysteine
TGF-β	Transforming growth factor beta
TLR	Toll-like receptor (identified specifically as Toll-like receptor 4 in the text)
TNF-α	Tumor necrosis factor alpha
VSMCs	Vascular smooth muscle cells
WHO	World Health Organization
